# Micro‒Global Positioning Systems for Identifying Nightly Opportunities for Marburg Virus Spillover to Humans by Egyptian Rousette Bats

**DOI:** 10.3201/eid2911.230362

**Published:** 2023-11

**Authors:** Brian R. Amman, Amy J. Schuh, Gloria Akurut, Kilama Kamugisha, Dianah Namanya, Tara K. Sealy, James C. Graziano, Eric Enyel, Emily A. Wright, Stephen Balinandi, Julius J. Lutwama, Rebekah C. Kading, Patrick Atimnedi, Jonathan S. Towner

**Affiliations:** Centers for Disease Control and Prevention, Atlanta, Georgia, USA (B.R. Amman, A.J. Schuh, T.K. Sealy, J.C. Graziano, J.S. Towner);; Uganda Wildlife Authority, Kampala, Uganda (G. Akarut, K. Kamugisha, D. Namanya, E. Enyel, P. Atimnedi);; Oak Ridge Institute of Science and Education, Oak Ridge, Tennessee, USA (E.A. Wright);; Uganda Virus Research Institute, Entebbe, Uganda (S. Balinandi, J.J. Lutwama);; Colorado State University, Fort Collins, Colorado, USA (R.C. Kading)

**Keywords:** Marburg virus, Ravn virus, Filovirus, viruses, Egyptian rousette bat, Rousettus aegyptiacus, bats, natural reservoir, spillover, humans, Marburg virus disease, nightly opportunities, global positioning system, tracking, zoonoses, Uganda

## Abstract

Marburg virus disease, caused by Marburg and Ravn orthomarburgviruses, emerges sporadically in sub-Saharan Africa and is often fatal in humans. The natural reservoir is the Egyptian rousette bat (ERB), which sheds virus in saliva, urine, and feces. Frugivorous ERBs discard test-bitten and partially eaten fruit, potentially leaving infectious virus behind that could be consumed by other susceptible animals or humans. Historically, 8 of 17 known Marburg virus disease outbreaks have been linked to human encroachment on ERB habitats, but no linkage exists for the other 9 outbreaks, raising the question of how bats and humans might intersect, leading to virus spillover. We used micro‒global positioning systems to identify nightly ERB foraging locations. ERBs from a known Marburg virus‒infected population traveled long distances to feed in cultivated fruit trees near homes. Our results show that ERB foraging behavior represents a Marburg virus spillover risk to humans and plausibly explains the origins of some past outbreaks.

Marburg virus (MARV) and Ravn virus (RAVV) (family Filoviridae, genus and species *Orthomarburgvirus marburgense*) are the causative agents of Marburg virus disease (MVD). The prototypical filovirus and close relative of Ebola virus, MARV was identified in Germany and the former Yugoslavia in 1967 when laboratory workers had MVD develop after handling infected African green monkeys (Cercopithecidae: *Chlorocebus tantalus*) imported from Uganda (*1*,*2*). Since then, 16 additional MVD outbreaks have been reported, with case-fatality rates of 23%‒90% (*3*). A reported large MVD outbreak in Africa (>100 cases) occurred at the Gorumbwa mine in eastern Democratic Republic of the Congo during 1998‒2000, followed nearly a decade later by 2 small outbreaks, at the Kitaka mine in southwest Uganda in 2007 (*4*,*5*) and Python Cave in Queen Elizabeth National Park, ≈32 km from Kitaka mine, in 2008 (*6*).

Subsequent ecologic investigations identified the Egyptian rousette bat (ERB; *Rousettus aegyptiacus*), a cave-dwelling fruit bat, as the MARV natural reservoir based on the repeated detection or isolation of MARV and RAVV directly from ERBs captured at those and other locations (*7**–**15*). This conclusion is further supported by experimental infection studies showing that ERBs are capable of shedding MARV for up to 3 weeks, the highest amounts in oral secretions and urine and to a lesser extent in feces (*16**–**18*), and that sustained bat-to-bat MARV transmission is possible under laboratory conditions in the absence of any other animals or arthropods normally found in their natural habitat (*18*).

Many MVD outbreaks were associated with direct human encroachment into ERB roosts and presumably exposure to infectious ERB material such as urine or feces. However, more than half (9/17) of historic MARV spillover events are epidemiologically unlinked to ERB cave habitats or infected secondary hosts. Because ERBs in Africa prefer to roost in unpopulated forested areas, pinpointing the intersections between ERBs and humans is difficult. Moreover, human infection with MARV occurs through broken skin or mucous membranes such as the eyes, nose, or mouth, implying the need for direct or indirect contact with an infected bat or infectious bodily fluids.

The question remains then, how do MVD outbreaks that are geographically distant from and epidemiologically unlinked to caves or mines get started? One possibility is suggested by virus stability studies showing that MARV remains viable for up to 5 days on surfaces such as wool and glass (*19*) or up to 6 hours on the surfaces of banana and mango, favorite foods among wild ERBs (*20*). This level of virus stability might provide sufficient time for humans, nonhuman primates (NHPs), or other potentially susceptible wildlife or domestic animals to be infected by indirect contact with contaminated surfaces or by consumption of fruits soiled with infectious ERB urine, feces, or saliva. ERBs routinely test bite and discard unwanted fruit or spit out masticated fruit pulp in the tree or on the ground (*21*,*22*), providing another means for releasing infectious virus into the environment. This behavior of test biting fruit most likely extends to all ERBs throughout their distribution in sub-Saharan Africa, where multiple other MVD outbreaks have occurred (Figure 1). We used micro‒global positioning systems (micro-GPS) to track nightly movements of ERBs in Uganda to identify opportunities for MARV spillover to humans.

**Figure 1:**
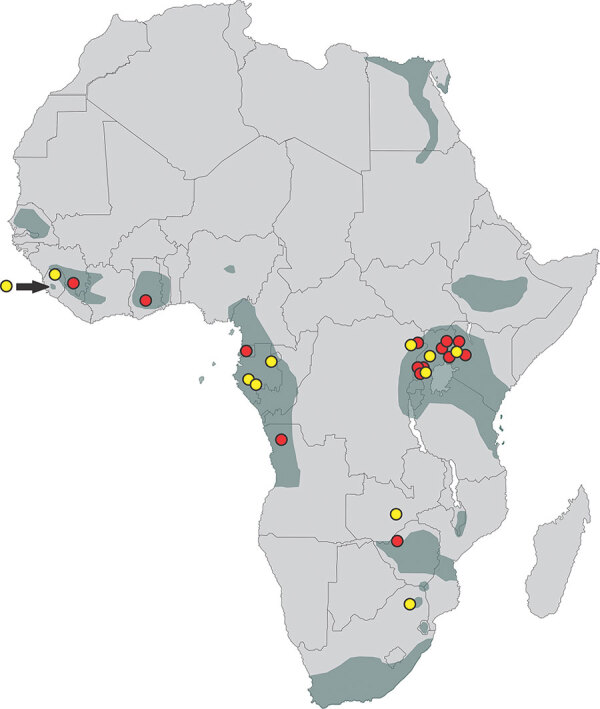
Distribution of *Rousettus aegyptiacus* bats in Africa (dark gray shading), showing locations of known Marburg virus disease spillover events into humans (red dots) and Egyptian rousette bats (*R. aegyptiacus*) that previously tested positive for Marburg or Ravn viruses (yellow dots). The bat distribution was adapted from the International Union for Conservation of Nature and Natural Resources Red List of Threatened and Endangered Species distribution maps (https://www.iucnredlist.org), except for the shaded area in Sierra Leone indicated by the yellow dot and black arrow, which represents a range extension for Egyptian rousette bats not shown on the Red List website (*7*).

## Methods

### Ethics and Biosafety

All animal work was performed with approval of the Centers for Disease Control and Prevention Institutional Animal Care and Use Committee (protocol no. 3063AMMBATX-A2) and with permissions from the Uganda National Counsel for Science and Technology (NS 614) and the Uganda Wildlife Authority (COD96/05) and through collaborations with Uganda Wildlife Authority and Uganda Virus Research Institute. All personnel wore appropriate personal protective equipment while performing all bat handling aspects of this field study as described (*23*). Those materials and procedures included disposable gowns or Tyvek coveralls, double gloves (including bite-resistant gloves if necessary), face shields, and respiratory protection.

### Bat Capture and Processing

To determine if ERBs, originating from a known MARV-infected population, routinely travel long distances to forage in cultivated fruit crops near homes, we fitted 100 male bats from Python Cave in Maramagambo Forest, part of Queen Elizabeth National Park in Uganda, with micro-GPS units and tracked their nightly movements. This process was performed over 2 separate capture sessions, once in February 2022 and again in August 2022, marking 50 bats each time. We selectively captured the bats by using handheld sweep nets. Only adult male bats were used in this study, to avoid burdening pregnant female bats with the GPS units.

As part of an ongoing zoonotic virus surveillance effort at Python Cave, an additional 50 bats were captured for destructive sampling. The bats were humanely euthanized under anesthesia and necropsied following procedures outlined in Amman et al. (*23*). Tissues harvested were cardiac blood, liver, spleen, heart, lung, kidney, salivary gland, and axillary lymph node; tissues were either flash frozen in liquid nitrogen for storage or placed in of virucidal lysis buffer and homogenized (100 mg tissue in 1 mL MagMax Lysis Buffer; Life Technologies, https://www.thermofisher.com) for RNA extraction and downstream PCR analysis. When handling bats and performing necropsies, all personnel wore appropriate personal protective equipment that included disposable gowns, double gloves )including bite-resistant gloves if necessary), and powered air-purifying respiratory protection.

### Viral Detection

We extracted RNA from 125 µL of tissue homogenate on the MagMAX Express-96 Deep Well Magnetic Particle Processor (Thermo Fisher Scientific, https://www.thermofisher.com) from homogenized tissues by using the MagMAX Total RNA Isolation Kit (Thermo Fisher Scientific). We then tested the extracted RNA by quantitative reverse transcription PCR (qRT-PCR) targeting the MARV protein 40 gene (VP40; see Amman et al. [*7*] for primer and probe sequences) and the eukaryotic 18S rRNA gene (Eukaryotic 18S rRNA Endogenous Control Kit; Thermo Fisher) by using the SuperScript III Platinum One-Step qRT-PCR Kit (Thermo Fisher Scientific).

### GPS Tracking and Data Analysis

**Figure 2:**
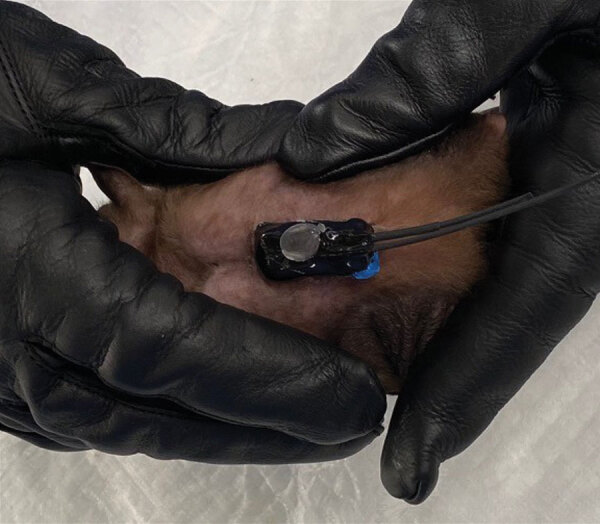
Micro‒global positioning system placement on an adult male Egyptian rousette bat (*Rousettus aegyptiacus*). Micro‒global positioning system units (<7 g; Telemetry Solutions, https://www.telemetrysolutions.com) were attached to the dorsum of male bats weighing >100 g.

We attached small (<7 g) micro-GPS units (Telemetry Solutions, https://www.telemetrysolutions.com) to an area of the bat’s dorsum just between the scapulae (Figure 2) by using a veterinary adhesive (America’s Acres Inc., https://americasacres.com). We used male bats weighing >100 g (mean 161.0 g, range 118.0–188.0 g) to keep the total percentage of unit weight to bodyweight ratio <10%. We kept bats fitted with GPS units in a screenhouse for several minutes to ensure a good GPS fit and unincumbered flight before release. The GPS units were preprogramed to begin collecting data at 5-minute intervals beginning at 7:00 pm and stopping at 5:00 am the next morning for the duration of the battery life. We placed a base station capable of wireless data download (Telemetry Solutions) just inside the entrance of Python Cave so that the entire cave interior was within line-of-site. To preserve GPS battery life, we used a repeater antenna outside the cave to transmit satellite signal to the GPS units on the bats inside the cave.

We collected the base station 5–7 days after the last GPS unit was deployed and downloaded the data to the Collar software (Telemetry Solutions) where it was converted to KML (Keyhole Markup Language) file format and viewed on Google Earth Pro version 7.3.4.8642 (https://www.google.com) to locate foraging sites and generate minimum convex polygons. We calculated average flight distance by using ArcGIS Pro 2.9.2 (Environmental Systems Research Institute, Inc., https://www.esri.com) to estimate movement by converting multiple KML layers to shapefiles and using the Generate Near Table Tool (https://pro.arcgis.com) with the Geodesic method selected for the Method parameter to calculate distances between the furthest 2 GPS locations. We defined foraging sites as areas where the GPS-fitted bats remained in the area, presumably feeding, for >30 minutes. We examined GPS trajectories to locate bat visits within 0–30 m from houses, cultivated crops, or forest patches near farms or cultivated crops outside the contiguous forest preserve.

## Results

### MARV Circulation in ERBs

Analysis of samples acquired from the ongoing viral zoonoses surveillance has shown that MARV continues to circulate in the ERB population roosting in Python Cave. Of the 50 bats captured for destructive sampling, 2 (4.0%) of 50 had detectable MARV RNA identified by qRT-PCR.

### GPS Tracking

Of the 100 ERBs fitted with GPS units, data from 70 bats were ultimately usable. Data from the other 30 units either were never received or were not usable because the data points were so few that no discernable flight pattern could be determined. We suspect that complete GPS failures were caused by the unit being pulled off by the bat or its roost mates or by the bat not returning to that roost, preventing the wireless download of data to the base station. Other reasons might include failure of the unit, including the battery; failure of the adhesive; or predation by one of the many snakes living in the cave or some other predator, such as birds of prey, in the surrounding area. 

Plotting the bat GPS coordinates onto satellite imagery showed that over a maximum battery life span of 4 days (range 1.5–4 days), ERBs from Python Cave had an average flight distance of 60.18 km (range 0.83–1,176.10 km) and visited 6 different homes and 34 independent cultivated crop localities in the surrounding agricultural communities. In some instances, bats visited the same location on consecutive nights. In total, 68/70 bats with GPS data (97.14%) made at least 1 visit to a house, a cultivated crop, or a forest patch near a farm or home in an agricultural area outside of the forest preserve surrounding Python Cave.

**Figure 3:**
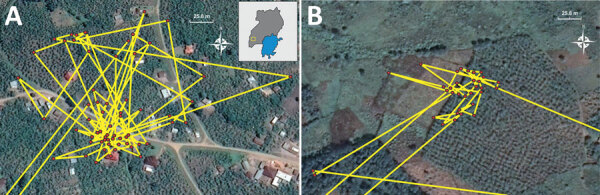
Google Earth Pro https://www.google.com/earth/versions) images of Egyptian rousette bat (*Rousettus aegyptiacus*) foraging activity, southwest Uganda, February 2022. Both images show micro‒global positioning system locations of Egyptian rousette bats from a population of known Marburg virus‒infected bats foraging in fruiting trees and cultivated crops near homes in southwest Uganda. Red dots indicate individual global positioning system points taken at 5-minute intervals, connected by yellow lines indicating the track from one point to another. A) Home with mango, avocado, papaya, and banana crops ≈12 km northeast of Python Cave visited by bat MV19. Total time spent at this site was 5 hours and 35 minutes (10:35 pm‒4:10 am) overnight on February 12, 2022. Inset map shows study area in Uganda (yellow square). B) Banana crop visited by bat MV18 200 m northwest of a farm, ≈49 km south-southwest of Python Cave. Total time spent at this site was 1 hour and 30 minutes 02:00 am–3:30 am) on February 13, 2022, and again for 1 hour and 20 minutes (2:40 am–4:00 am) on February 14, 2022.

**Figure 4:**
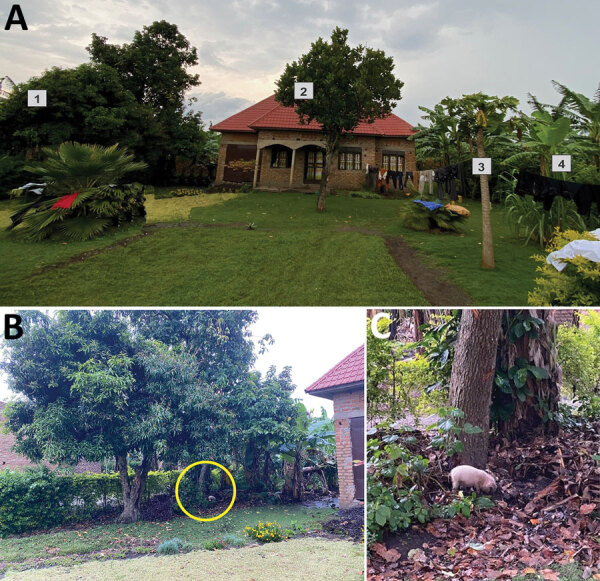
House visited by Egyptian rousette bat (*Rousettus aegyptiacus*) MV19, southwest Uganda, February 2022. A) Front view of the house where bat MV19 spent 10:35 pm‒4:10 am on February 12 and 13, 2022, foraging: 1, mango tree; 2, avocado tree; 3, papaya tree; 4, banana crop. B) Pig rooting underneath a mango tree (yellow circle). C) Enlargement of yellow circled area from panel B.

We assessed the accuracy of the GPS data by performing ground truthing, a method used to compare data from field measurements to data collected remotely, at 2 locations in August 2022 at sites where bats visited, presumably foraging, for extended periods on consecutive nights. At 1 house, bat MV19 visited for 5 hours and 35 minutes (10:35 pm‒4:10 am) overnight on February 12–13, 2022 (Figure 3, panel A). There, mango, papaya, avocado, and bananas were directly observed growing in the yard and along the sides of the house (Figure 4, panel A). Furthermore, a young pig (*Sus scrofa*) was observed rooting under the mango tree (Figure 4, panel B, C). An interview with the homeowner confirmed that the mango tree was fruiting in February 2022 when the GPS-tagged bat foraged in the tree. At a second site, bat MV18 spent 1 hour and 30 minutes (2:00 am–3:30 am) on February 13, 2022, and then foraged again at the same site for 1 hour and 20 minutes (2:40 am–4:00 am) the following night (Figure 3, panel B). The crops visited at this second site were predominantly bananas but also mangos. We have not published GPS coordinates for those locations to protect the privacy of the property owners. 

### Movement Area

**Figure 5:**
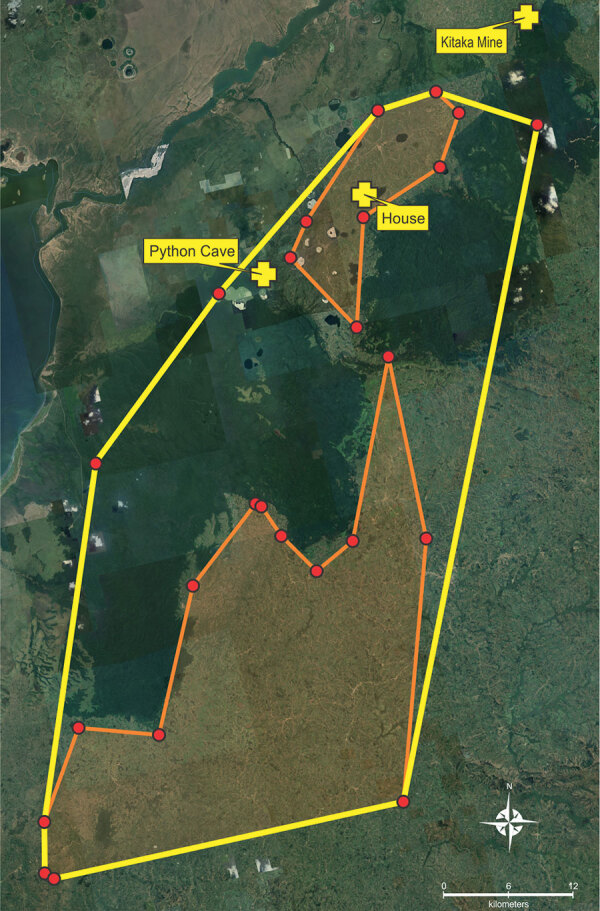
Minimum convex polygons (MCP) of Egyptian rousette bat (*Rousettus aegyptiacus*) total forage area, southwest Uganda, February and August 2022. Yellow lines indicate MCP of forage area overall (1,887 km^2^); orange lines indicate MCP of forage area in agricultural communities (846 km^2^). Red dots for all MCPs indicate the outermost peripheral sites where the bats fitted with micro‒global positioning system units foraged for >30 minutes. MCPs are shown in relation to Python Cave, where the Egyptian rousette bats roost and were fitted with the micro‒global positioning system; the house visited by bat MV19; and Kitaka Mine, the site of a 2007 Marburg virus outbreak.

To quantify the estimated minimum movement area where the ERBs appeared to forage nightly, we created a minimum convex polygon (MCP) (*24*–*27*) by using the outermost peripheral sites (n = 9) where the GPS-fitted bats foraged for >30 minutes (homes, crops, and forest patches) for both February and August 2022. In total, the area of bat foraging activity encompassed by this MCP was 1,887 km^2^, of which 44.8% (846 km^2^) was in agricultural or community areas outside the forest (Figure 5). Even though the shortest distance from Python Cave and the forest preserve to an agricultural area was just >0.5 km, the longest distance traveled by a single bat in 1 night was 57 km, suggesting a theoretical foraging zone radiating >50 km in any direction from an ERB roost. The GPS data also showed that several ERBs came within 10 km of Kitaka mine, where, in 2007, 2.8% of an ERB colony containing >100,000 animals, or ≈5,000 bats total, were found to be actively infected with either MARV or RAVV (*13*).

## Discussion

Analysis of the tissues from the bats euthanized as part of ongoing zoonotic viral surveillance determined that MARV continued to circulate in Python Cave almost 15 years after 2 tourists were infected there, once in December 2007 and again in July 2008 (*6*,*28*). Moreover, the level of active infection was consistent with that found among adult bats during previous studies at Python Cave (*8*), albeit with a much smaller sample size. The data reported showed that ERBs at Python Cave, a forest-bound roost of >40,000 bats (*8*), routinely travel distances up to 57 km to forage in peripheral agricultural areas where they have easy access to human cultivated fruits and wild fruiting trees opportunistically used by humans and domestic livestock. Given the known distribution of ERBs in Africa, overlayed by locations of past MVD outbreaks and MARV-positive ERBs (Figure 1), the potential area in Africa at risk for virus spillover is considerable but, on a fine scale, might be limited to foraging range of ERB roosts. This behavior presents a previously underappreciated geographic range and potential mechanism of MARV spillover that might explain the origins of MVD outbreaks, such as those most recently in Guinea in 2021, Ghana in 2022, and Equatorial Guinea and Tanzania in 2023. In all instances, ERBs were reported near the index case, but ensuing epidemiologic investigations produced no links between the primary cases and known ERB colonies. 

At Python Cave, 6 of the 70 GPS-tagged ERBs foraged in fruiting trees near houses, sometimes on consecutive nights by the same bat; 1 house was later confirmed to have both mangoes and bananas growing within a few meters of the structure. In that instance, the ERB stayed at the location for >5.5 hours, vacating the area just 2 hours before sunrise, leaving a period of up to 4 hours after sunrise for humans to encounter infectious virus shed in ERB excreta or oral secretions.

The role of secondary animal hosts must also be considered, and at the time of ground truthing, a young pig was observed rooting under the same mango tree the ERB visited, not far from children playing in the yard. Pigs are known to be susceptible to ebolavirus (Reston and Ebola virus) infection (*29*,*30*), which suggest a potential susceptibility to MARV infection. Given that experimental infection studies of ERBs have shown that >10^4^ 50% tissue culture infectious doses/mL of MARV can be shed intermittently in oral secretions for up to 19 days after infection (*16*,*18*,*31*), this mechanism of bat-to-human MARV transmission is plausible, as well as potential transmission events involving intermediate hosts such as pigs or NHPs. Such competition for cultivated fruit could draw ERBs and other animals together in time and space when they otherwise might not interact.

MVD and Ebola disease outbreaks have been initiated through hunting or scavenging of infected sick, dying, or recently dead animals or by unwitting importation of diseased NHPs for research (*1*,*2*,*32*–*34*). Both Reston virus, an ebolavirus, and Nipah virus, a paramyxovirus carried by multiple species of *Pteropus* bats, have emerged in pigs (*35*,*36*), and Reston virus emerged in NHPs imported from the Philippines to the United States in 1989 (*37*). Moreover, domestic pigs were believed to have been infected with Nipah virus by eating fruit dropped by bats that was contaminated with bat excreta (*38*), and infectious MARV was recovered from experimentally inoculated banana and mango up to 6 hours postinoculation (*20*).

Although the exact number of bats visiting the areas identified by the GPS units is unknown, newly independent juvenile ERBs have been shown to use the same navigation routes and foraging areas as their mothers (*39*). Therefore, it is probable that older juvenile bats, the age cohort with the highest incidence of MARV infection (*8*), forage on the same cultivated crops as their male GPS-fitted ERB counterparts. This result is supported by findings that human-generated land use changes are linked to increases in the abundance of zoonotic reservoir species, along with decreases in nonreservoir species (*40*). This loss in biodiversity correlates with increased human exposure to zoonotic pathogens (*41*–*43*). It is therefore not difficult to picture the remnants of a forest surrounded by drastically altered agricultural areas as having much reduced biodiversity, leading to increased prevalence of ERBs practicing foraging patterns learned from older ERBs, thereby continuing to circulate MARVs in the surrounding altered habitat areas.

The GPS data showed that several ERBs came close to Kitaka mine, which supports earlier published accounts that infected ERBs at Python Cave, ≈32 km from Kitaka mine, routinely travel between roosts, effectively creating a small scale metapopulation within 2 forest preserves (*8*). Combined with twice yearly breeding and seasonal pulses of up to 12% MARV infection in juvenile ERBs, this finding could help explain how such a large amount of MARV genetic diversity is maintained at those 2 locations (*8*,*13*,*44*). The foraging data also suggest that other homes and agricultural areas in Uganda, beyond what is shown here, are frequented by ERBs. To get a sense of the overall potential for ERB-human interactions, we can consider that 8.5% (6/70) marked bats visited fruit trees near homes over an ≈4-day period. Extending those values to an ERB population of 40,000 bats, 2.8% of which are actively infected, translates to 95 home visits by MARV-infected bats every 4 days, or 8,668 infected bat visits per year. Although highly consequential if infection does occur, those numbers also suggest that, despite the large geographic zone of risk surrounding ERB roosts, actual spillover events that result in outbreaks are rare, probably requiring an alignment of multiple circumstances, including seasonal fruiting periods, akin to that proposed for how Hendra virus spills over into horses (*45*). Nevertheless, a successful transmission event of this batborne virus or others, even if extremely rare, can be devastating. Examples include the recent Ebola outbreak in West Africa during 2013–2016 that infected >28,000 persons, primarily in Guinea, Sierra Leone, and Liberia, and, more recently, the COVID-19 pandemic caused by SARS-CoV-2, a batborne coronavirus that spilled over into humans, possibly from a horseshoe bat (*Rhinolophus affinis*) (*46*).

We report findings of visits to cultivated fruit crops near homes and farms by a known MARV natural reservoir, the ERB. Although this information has public health usefulness, the study has several limitations, including battery limitations of the GPS units, shorter time frames for recording foraging activity, and that only male bats have been fitted with GPS units to this point in the study. Investigations of female and older juvenile bats >100 g, which are the more actively infected cohort of the population (*8*), will be useful. Longer monitoring periods with fewer data points collected to preserve battery life and increase the number of days the bats will be tracked will also be useful. 

If one considers the longevity of MARV on surfaces and fruit commonly consumed by humans in Uganda and the propensity of the natural reservoir to use human cultivated fruit crops as a source of food, it is conceivable that surface contamination of fruits and other surfaces around farms and homes is the mechanism by which some previous MVD outbreaks unlinked to ERB roosts were initiated. The data we collected will be developed into a larger, evidence-based risk map of MARV spillover in Uganda. Public health messaging for at-risk communities will include the need for properly washing harvested fruits, discarding partially eaten fruits, and keeping livestock away from fallen fruit. Furthermore, this type of evidence-based risk mapping can be used as a rationale for conducting enhanced surveillance activities at local health clinics servicing at-risk communities, including active mortality rate surveillance, to ensure rapid detection of spillover events when they occur.
